# Morphological and molecular identification of two new *Ganoderma* species on *Casuarina equisetifolia* from China

**DOI:** 10.3897/mycokeys.34.22593

**Published:** 2018-06-07

**Authors:** Jia-Hui Xing, Yi-Fei Sun, Yu-Li Han, Bao-Kai Cui, Yu-Cheng Dai

**Affiliations:** 1 Institute of Microbiology, Beijing Forestry University, Beijing 100083, China

**Keywords:** Ganodermataceae, medicinal mushroom, morphology, phylogeny, taxonomy, wood-rotting fungi

## Abstract

*Ganoderma* is a cosmopolitan white rot fungal genus, famous for its medicinal properties. In the present study, two new *Ganoderma* species were collected from south-eastern China and described on the basis of morphological characters and phylogenetic analyses of sequences of the internal transcribed spacer (ITS) region, the translation elongation factor 1-α gene (EF1-α) and the second subunit of RNA polymerase II (RPB2). Specimens of both species were found on living trees of *Casuarina equisetifolia*. *Ganoderma angustisporum* **sp. nov.** is characterised by its sessile basidiomata and almond-shaped, slightly truncate, narrow basidiospores (9–11.3 × 4–5.2 µm). *Ganoderma casuarinicola* **sp. nov.** is characterised by its strongly laccate reddish-brown pileal surface, luminous yellow to yellowish-brown cutis and ellipsoid, truncate basidiospores (9–10.2 × 5–6 µm). The two new species are compared with their related taxa. Phylogenetic analyses confirmed that *G. angustisporum* and *G. casuarinicola* are distinct species within *Ganoderma*.

## Introduction

*Ganoderma* P. Karst. is easily recognised by its characteristic appearance, double-walled and truncate basidiospores (Karsten 1881; Moncalvo and Ryvarden 1997). According to Christian (2015), there are 428 names in the Index Fungorum (http://www.indexfungorum.org/) and 456 records of taxa (420 with status legitimate) in MycoBank (http://www.mycobank.org/). *Ganoderma* products as dietary supplement are very popular in Asia, especially in China and China is very rich in *Ganoderma* species (Zhao and Zhang 2000; Wang et al. 2009; Cao et al. 2012; Cao and Yuan 2013; Li et al. 2014b). The great variability in macroscopic characters of basidiomes has resulted in a large number of synonyms and confusions in the taxonomy of this genus. Using DNA sequence data for the identification of *Ganoderma* species is of greatest importance.

Some *Ganoderma* species are well known for causing wood decay in a wide range of tree species around the world. For example, *G. boninense* Pat. is a causal agent of oil palm basal stem rot and is responsible for considerable yield losses in southeast Asian oil palm plantations (Pilotti 2005). Especially in Indonesia and Malaysia, *G. boninense* and *G. philippii* (Bres. & Henn. ex Sacc.) Bres. cause great economic loss of palm oil, tea and rubber (Zakaria et al. 2009).

*Casuarina equisetifolia* Forst. is used as an industrial raw material and wood fuel, as well as for conservation of coastal ecosystems and for agricultural land protection against salinity intrusion (Hossain et al. 1998; Chowdhury et al. 2009). In China, *Casuarina equisetifolia* is widely planted in the coastal areas of Guangxi, Guangdong, Fujian, Hainan and Taiwan provinces. During collections of wood-rotting fungi in South China in recent years, two *Ganoderma* species growing on *Casuarina equisetifolia*, which could not be identified to any known species, were collected. Those two species are here described based on morphological characters and phylogenetic analyses.

## Materials and methods

### Morphological studies

The examined specimens were deposited in the herbarium of the Institute of Microbiology, Beijing Forestry University (BJFC). Macro-morphological descriptions were based on field notes. Special colour terms followed Petersen (1996). Micro-morphological data were obtained from the dried specimens and observed under a light microscope following Li et al. (2014a) and Han et al. (2016). Sections were studied at a magnification of up to 1000× using a Nikon E 80i microscope and phase contrast illumination. Drawings were made with the aid of a drawing tube. Microscopic features, measurements and drawings were made from slide preparations stained with Cotton Blue and Melzer’s reagent. Spores were measured from sections cut from the tubes. To represent variation in the size of basidiospores, 5% of measurements were excluded from each end of the range and extreme values are given in parentheses.

The following abbreviations are used: IKI = Melzer’s reagent, IKI– = neither amyloid nor dextrinoid, KOH = 5% potassium hydroxide, CB = Cotton Blue, CB+ = cyanophilous, Q is an average computed by dividing the length by the width of each spore separately, *n* (*a*,*b*) = *a* spores measured from *b* specimens.

### Molecular study and phylogenetic analysis

The CTAB rapid plant genome extraction kit-DN14 (Aidlab Biotechnologies Co. Ltd., Beijing, China) was used to extract total genomic DNA from dried specimens according to the manufacturer’s instructions with some modifications (Chen et al. 2016, 2017). The genes ITS, EF1-α and RPB2 were amplified by polymerase chain reaction (PCR) technique. The ITS region was amplified with primer pair ITS5 (GGA AGT AAA AGT CGT AAC AAG G) and ITS4 (TCC TCC GCT TAT TGA TAT GC) (White et al. 1990). Part of the EF1-α gene was amplified with primer pair EF1-983F (GCY CCY GGH CAY CGT GAY TTY AT) and EF1-1567R (ACH GTR CCR ATA CCA CCR ATC TT) (Rehner and Buckley 2005). Part of the RPB2 gene was amplified with primer pairs 5F (GAY GAY MGW GAT CAY TTY GG) and 7CR (CCC ATR GCT TGY TTR CCC AT) (Liu et al. 1999). The PCR cycling for ITS was as follows: initial denaturation at 95 °C for 3 min, followed by 35 cycles at 94 °C for 40 s, 54 °C for 45 s and 72 °C for 1 min and a final extension of 72 °C for 10 min. The PCR cycling for EF1-α was as follows: initial denaturation at 95 °C for 3 min, followed by 34 cycles at 94 °C for 40 s, 56 °C for 45 s and 72 °C for 1 min and a final extension of 72 °C for 10 min. The PCR cycling for RPB2 was as follows: initial denaturation at 95 °C for 5 min, followed by 35 cycles at 95 °C for 1 min, 58 °C for 2 min and 72 °C for 1.5 min and a final extension of 72 °C for 10 min. The PCR products were purified and sequenced at Beijing Genomics Institute (China), using forward and reverse PCR primers. All newly generated sequences were deposited in GenBank (Table 1).

**Table 1.** Species, specimens, geographic origin and GenBank accession numbers of sequences used in this study.

Species name	Voucher no.	Geographic origin	GenBank accession numbers			References
			ITS	EF1-α	RPB2	
***Ganoderma angustisporum***	Cui 13817 (holotype)	Fujian, China	MG279170*	MG367563*	MG367507*	this study
***G. angustisporum***	Cui 14578	Guangdong, China	MG279171*	MG367564*	–	this study
***G. angustisporum***	Cui 16340	Guangxi, China	MG279172*	–	–	this study
*G. aridicola*	Dai 12588 (holotype)	Durban, South Africa	KU572491	KU572502	–	Xing et al. 2016
*G. boninense*	WD 2028	Japan	KJ143905	KJ143924	KJ143964	Zhou et al. 2014
*G. boninense*	WD 2085	Japan	KJ143906	KJ143925	KJ143965	Zhou et al. 2014
***G. casuarinicola***	Dai 16336 (holotype)	Guangdong, China	MG279173*	MG367565*	MG367508*	this study
***G. casuarinicola***	Dai 16337	Guangdong, China	MG279174*	MG367566*	MG367509*	this study
***G. casuarinicola***	Dai 16338	Guangdong, China	MG279175*	MG367567*	MG367510*	this study
***G. casuarinicola***	Dai 16339	Guangdong, China	MG279176*	MG367568*	MG367511*	this study
*G. curtisii*	CBS 100131	NC, USA	JQ781848	KJ143926	KJ143966	Zhou et al. 2014
*G. curtisii*	CBS 100132	NC, USA	JQ781849	KJ143927	KJ143967	Zhou et al. 2014
*G. destructans*	CBS 139793 (type)	Pretoria, South Africa	NR132919	–	–	Coetzee et al. 2015
*G. destructans*	CMW 43670	Pretoria, South Africa	KR183856	–	–	Coetzee et al. 2015
*G. destructans*	Dai 16431	South Africa	MG279177*	MG367569*	MG367512*	this study
*G. enigmaticum*	CBS 139792 (type)	Pretoria, South Africa	NR132918	–	–	Coetzee et al. 2015
*G. enigmaticum*	Dai 15970	Africa	KU572486	KU572496	MG367513*	Xing et al. 2016; this study
*G. enigmaticum*	Dai 15971	Africa	KU572487	KU572497	MG367514*	Xing et al. 2016; this study
*G. heohnelianum*	Dai 11995	Yunnan, China	KU219988	MG367550*	MG367497*	Song et al. 2016b; this study
*G. heohnelianum*	Yuan 6337	Guangxi, China	MG279160*	MG367551*	MG367498*	this study
*G. heohnelianum*	Cui 13982	Guangxi, China	MG279178*	MG367570*	MG367515*	this study
*G. leucocontextum*	GDGM 44489	Xizang, China	KM396271	–	–	Li et al. 2014b
*G. leucocontextum*	GDGM 44490	Xizang, China	KM396272	–	–	Li et al. 2014b
*G. leucocontextum*	Dai 15601	Xizang, China	KU572485	KU572495	MG367516*	Xing et al. 2016; this study
*G. lingzhi*	Wu 1006-38 (holotype)	Hubei, China	JQ781858	JX029976	JX029980	Cao et al. 2012
*G. lingzhi*	Cui 14342	Sichuan, China	MG279179*	MG367571*	MG367517*	this study
*G. lingzhi*	Cui 14375	Sichuan, China	MG279180*	MG367572*	MG367518*	this study
*G. lobatum*	JV 1008/31	USA	KF605671	MG367553*	MG367499*	this study
*G. lobatum*	JV 1008/32	USA	KF605670	MG367554*	MG367500*	this study
*G. lucidum*	K 175217	UK, Europe	KJ143911	KJ143929	KJ143971	Zhou et al. 2014
*G. lucidum*	Cui 14404	Sichuan, China	MG279181*	MG367573*	MG367519*	this study
*G. lucidum*	Cui 14405	Sichuan, China	MG279182*	MG367574*	MG367520*	this study
*G. martinicense*	LIP SW-Mart08-44	Martinica	KF963257	–	–	Welti and Courtecuisse 2010
*G. martinicense*	LIP SW-Mart08-55 (type)	Martinica	KF963256	–	–	Welti and Courtecuisse 2010
*G. martinicense*	He 2240	USA	MG279163*	MG367557*	MG367503*	this study
*G. multipileum*	CWN 04670	Taiwan, China	KJ143913	KJ143931	KJ143972	Zhou et al. 2014
*G. multipileum*	Dai 9447	Hainan, China	KJ143914	–	KJ143973	Zhou et al. 2014
*G. multipileum*	Cui 14373	Sichuan, China	MG279184*	MG367575*	MG367521*	this study
*G. multiplicatum*	SPC9	Brazil	KU569553	–	–	Bolaños et al. 2016
*G. multiplicatum*	60119011	Brazil	MG279185*	–	–	this study
*G. multiplicatum*	URM 83346	Brazil	JX310823	–	–	Bolaños et al. 2016
*G. orbiforme*	Cui 13918	Hainan, China	MG279186*	MG367576*	MG367522*	this study
*G. orbiforme*	Cui 13880	Hainan, China	MG279187*	MG367577*	MG367523*	this study
*G. philippii*	Cui 14443	Hainan, China	MG279188*	MG367578*	MG367524*	this study
*G. philippii*	Cui 14444	Hainan, China	MG279189*	MG367579*	MG367525*	this study
*G. resinaceum*	Rivoire 4150	France, Europe	KJ143915	–	–	Zhou et al. 2014
*G. resinaceum*	CBS 194.76	Netherlands, Europe	KJ143916	KJ143934	–	Zhou et al. 2014
*G. ryvardenii*	HKAS 58053 (type)	Cameroon, Africa	HM138671	–	–	Kinge and Mih 2011
*G. ryvardenii*	HKAS 58054	Cameroon, Africa	HM138672	–	–	Kinge and Mih 2011
*G. ryvardenii*	HKAS 58055	Cameroon, Africa	HM138670	–	–	Kinge and Mih 2011
*G. shandongense*	Dai 15785	Shandong, China	MG279190*	MG367580*	MG367526*	this study
*G. shandongense*	Dai 15787	Shandong, China	MG279191*	MG367581*	MG367527*	this study
*G. shandongense*	Dai 15791	Shandong, China	MG279192*	MG367582*	MG367528*	this study
*G. sinense*	Wei 5327	Hainan, China	KF494998	KF494976	MG367529*	this study
*G. sinense*	Cui 13835	Hainan, China	MG279193*	MG367583*	MG367530*	this study
*G. tropicum*	He 1232	Guangxi, China	KF495000	MG367584*	MG367531*	this study
*G. tropicum*	Yuan 3490	Yunnan, China	JQ781880	KJ143938	–	Cao et al. 2012
*G. tropicum*	Dai 16434	Hainan, China	MG279194*	MG367585*	MG367532*	this study
*G. tsugae*	Dai 12751b	CT, USA	KJ143919	KJ143939	KJ143977	Zhou et al. 2014
*G. tsugae*	Cui 14110	Jilin, China	MG279195*	MG367586*	MG367533*	this study
*G. tsugae*	Cui 14112	Jilin, China	MG279196*	MG367587*	MG367534*	this study
*G. weberianum*	CBS 219.36	Philippines	JQ520219	–	–	Zhou et al. 2014
*G. williamsianum*	Wei 5032	Hainan, China	KU219994	–	–	Song et al. 2016b
*G. williamsianum*	Dai 16809	Thailand	MG279183*	MG367588*	MG367535*	this study
*G. zonatum*	FL-02	FL, USA	KJ143921	KJ143941	KJ143979	Zhou et al. 2014
*G. zonatum*	FL-03	FL, USA	KJ143922	KJ143942	KJ143980	Zhou et al. 2014
Outgroup						
*Amauroderma rugosum*	Cui 9011	Guangdong, China	KJ531664	KU572504	MG367506*	Li and Yuan 2015; this study
*Tomophagus colossus*	TC-02	Vietnam	KJ143923	KJ143943	–	Zhou et al. 2014

* Newly generated sequences for this study.

Bold names = new species.

Besides the sequences generated from this study, other reference sequences were selected from GenBank for phylogenetic analyses. Sequences were aligned in MAFFT 6 (Katoh and Toh 2008; http://mafft.cbrc.jp/alignment/server/) using the “G-INS-I” strategy and manually adjusted in BioEdit (Hall 1999). Sequence alignment was deposited in TreeBase (http://purl.org/phylo/treebase/phylows/study/TB2:S22403; submission ID 22403). *Amauroderma rugosum* (Blume & T. Nees) Torrend and *Tomophagus colossus* (Fr.) C.F. Baker were selected as outgroups.

Phylogenetic analyses in this study followed the approach of Song et al. (2016a) and Song and Cui (2017). The maximum likelihood (ML) and Bayesian inference (BI) methods were used to analyse the combined dataset of ITS, EF1-α and RPB2 sequences. ML analysis was conducted with RAxML-HPC252 on the Cipres Science Gateway (Miller and Pfeiffer 2011) involved 100 ML searches; all model parameters were estimated by the programme. The ML bootstrap values (ML-BS) were obtained with 1000 rapid bootstrapping replicates. BI was performed with MrBayes 3.1.2 (Ronquist and Huelsenbeck 2003), with a mixed model partition. A suitable substitution model for each partition of the dataset was determined using the Akaike Information Criterion implemented in MrMODELTEST 2.3. Four Markov chains were run from the random starting tree for 1 million generations to make the average standard deviation of split deviation frequencies lower than 0.01. Trees were sampled every 100 generations. The burn-in was set to discard the first 25% of the trees. A majority rule consensus tree of all the remaining trees was used to calculate Bayesian posterior probabilities (BPP). The ML and BI algorithms generated congruent topologies in main lineages; thus, only the topology from the ML algorithm was presented along with BS and BPP greater than 75% and 0.95, respectively, at the nodes.

## Results

### Phylogenetic analysis

The combined ITS, EF1-α and RPB2 dataset included sequences from 66 fungal samples representing 27 taxa. The selected models were K80 for 5.8S, K80 + G for ITS1, HKY + G for ITS2, GTR + I + G for ITS1+ ITS2 + 5.8S. The best model selected and applied in the BI analysis for the combined ITS, EF1-α and RPB2 partition was a GTR+I+G model. BI analysis and ML analysis resulted in the same topology with an average standard deviation of split frequencies = 0.006025 (BI).

### Taxonomy

#### Ganoderma angustisporum

Taxon classificationFungiPolyporalesGanodermataceae

J.H. Xing, B.K. Cui & Y.C. Dai sp. nov.

MB823320

Figs 2a–b , 3

##### Diagnosis.

*Ganoderma angustisporum* is characterised by its sessile basidiomata, white pore surface, almond-shaped, slightly truncate and narrow basidiospores.

##### Holotype.

CHINA. Fujian Prov., Pingtan County, on living tree of *Casuarina equisetifolia*, 18 August 2016, Cui 13817 (BJFC!).

##### Etymology.

*angustisporum* (Lat.): referring to the narrow basidiospores.

##### Description.

**Basidiomes** annual, sessile and broadly attached, applanate, shell-shaped, projecting up to 13.5 cm, 10 cm wide and 1.1 cm thick at base, corky when fresh, becoming hard corky to woody hard upon drying. Pileal surface strongly laccate, reddish-brown to dark brown, with a thin crust, concentrically zonate or azonate; margin distinct, slightly obtuse. Pore surface white when fresh, turning light buff when dry; pores round to angular, 3–5 per mm; dissepiments slightly thick to thick, entire. Context corky, homogeneous, greyish-brown, bearing distinct concentric growth zones, black melanoid band present, up to 0.4 cm thick. Tubes woody hard, greyish-brown, up to 0.7 cm long. **Hyphal system** trimitic; generative hyphae bearing clamp connections; all the hyphae IKI–, CB+; tissues darkening in KOH. **Pellis**: pellis cells regularly arranged into a palisade; terminal cells clavate, yellowish to pale brown, thin-walled, occasionally with blunt outgrowth and protuberance in the apical or lateral parts, bearing a simple septum at base, moderately amyloid at maturity, 15–33 × 4–10 μm. **Context** generative hyphae colourless, thin-walled, bearing clamp connections, unbranched, 2–4.5 µm in diam; skeletal hyphae dominant, pale yellowish-brown, thick-walled to subsolid, frequently branched, interwoven, 3–6 µm in diam; binding hyphae abundant, pale yellowish-brown, thick-walled with a narrow lumen to subsolid, frequently branched, tortuous, interwoven, 1–2.5 µm in diam. **Tubes** generative hyphae colourless, thin-walled, bearing clamp connections, unbranched, slightly swollen at the distal end, 2–2.8 μm in diam; skeletal hyphae dominant, pale brown to distinctly brown, thick-walled with a medium or narrow lumen to subsolid, frequently branched, strongly interwoven, 3–4.5 μm in diam; binding hyphae brownish-yellow, thick-walled to almost solid, frequently branched, interwoven, 1–1.8 μm in diam. Basidia barrel-shaped, yellowish to pale brown, with a clamp connection and four sterigmata, 11–16 × 6.5–9 µm; basidioles pear-shaped to fusiform, 8–15 × 5–8 µm. **Basidiospores** mostly almond-shaped at maturity, slightly truncate, yellowish to pale brown, IKI–, CB+, double-walled, exospore smooth, endospore with coarse echinulate, (8–)9–10.5(–11) × (3.5–)4–5 µm, L = 8.89 μm, W = 4.27 μm, Q = 2.01–2.24 (n = 60/2, with the turgid vesicular appendix excluded); (8–)9–11.3(–12) × (3.8–)4–5.2 µm, L = 10.26 μm, W = 4.31 μm, Q = 2.36–2.4 (n = 60/2, with the turgid vesicular appendix included).

**Figure 1.**
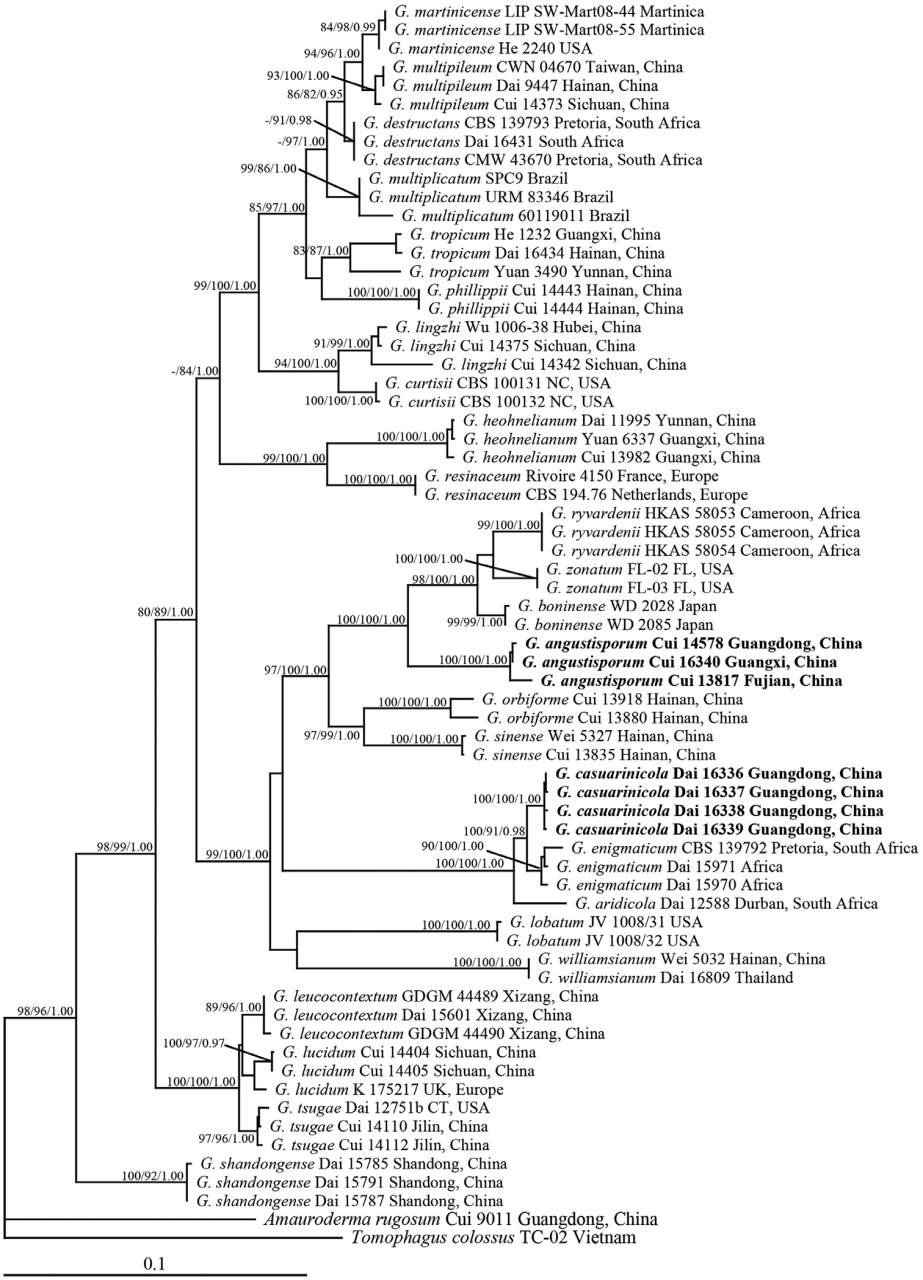
Phylogeny of the new *Ganoderma* species and related taxa based on ITS+EF1-α+RPB2 sequence data. Branches are labelled with bootstrap values (ML) higher than 75%, and posterior probabilities (BI) higher than 0.95. Bold names = new species.

**Figure 2.**
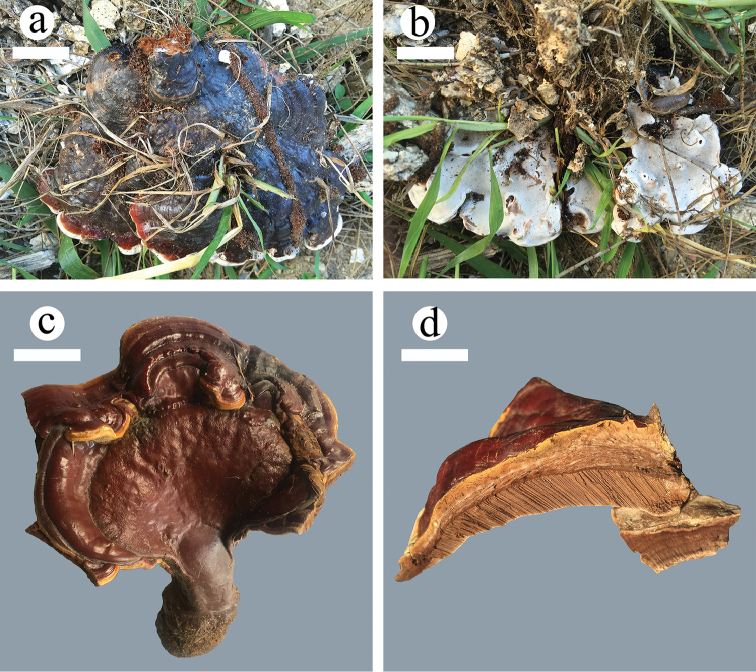
Basidiomata of *Ganoderma* species. **a, b** *G. angustisporum* (*Cui 13817*) **c, d** *G. casuarinicola* (*Dai 16336*). Scale bars: 2 cm.

**Figure 3.**
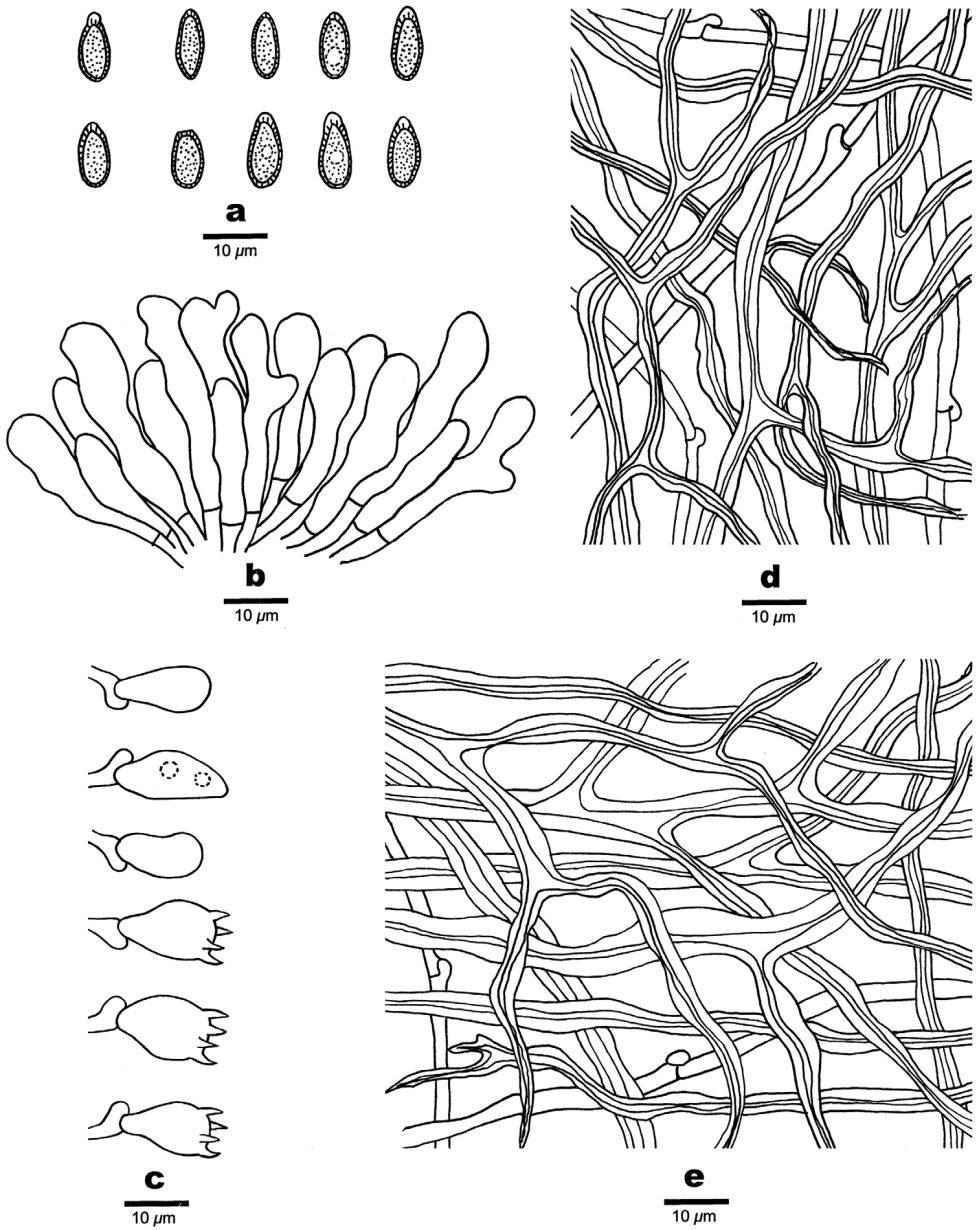
Microscopic structures of *Ganoderma angustisporum* (drawn from the holotype). **a** Basidiospores **b** Apical cells from the pellis **c** Basidia and basidioles **d** Hyphae from context **e** Hyphae from trama. Scale bars: 10 µm.

**Figure 4.**
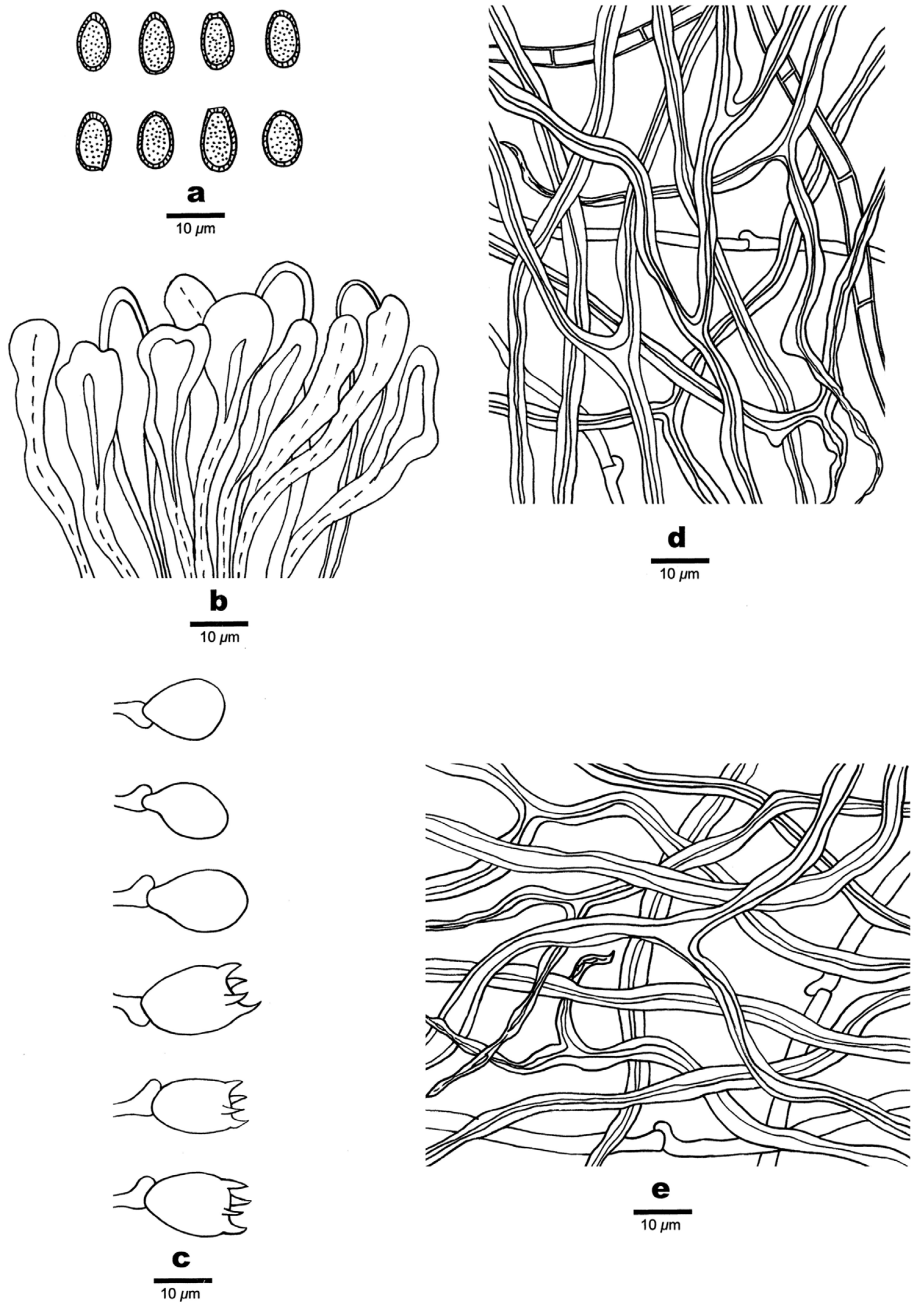
Microscopic structures of *Ganoderma casuarinicola* (drawn from the holotype). **a** Basidiospores **b** Apical cells from the cuticle **c** Basidia and basidioles **d** Hyphae from context **e** Hyphae from trama. Scale bars: 10 µm.

##### Type of rot.

A white rot.

##### Additional specimens examined.

CHINA. Guangdong Prov., Maoming, Dianbai, on living trees of *Casuarina equisetifolia*, 20 June 2017, Cui 14578, Cui 16494 and Cui 16495 (BJFC!).

#### Ganoderma casuarinicola

Taxon classificationFungiPolyporalesGanodermataceae

J.H. Xing, B.K. Cui & Y.C. Dai sp. nov.

MB823321

Figs 2c–d , 4

##### Diagnosis.

*Ganoderma casuarinicola* is characterised by its strongly laccate reddish-brown pileal surface, white pore surface, luminous yellow to yellowish-brown cutis.

##### Holotype.

CHINA. Guangdong Prov. Zhanjiang, Dianbai, on living tree of *Casuarina equisetifolia*, 4 October 2015, Dai 16336 (BJFC!).

##### Etymology.

***casuarinicola*** (Lat.): referring to the host tree genus *Casuarina*.

##### Description.

**Basidiomes** annual, stipitate to substipitate, pileus sectorial to shell-shaped, projecting up to 10 cm, 7 cm wide and 2 cm thick at base, corky, without odour when fresh, becoming hard corky to woody hard when dry. Pileal surface strongly laccate, reddish-brown, with a thin crust; margin obtuse, cream to reddish-brown. Stipe flattened or subcylindrical, lateral, reddish-brown, up to 6 cm long and 1.7 cm in diam. Pore surface white when fresh, turning cream when dry; pores round to angular, 4–6 per mm; dissepiments thin to slightly thick, entire. Context corky, heterogeneous, the upper layer generally light yellow up to 0.1 cm thick and the lower layer generally dark brown close to the tubes up to 1 cm thick, showing distinct concentric growth zones, black melanoid band absent. Tubes woody hard, greyish-brown, up to 0.9 cm long. **Hyphal system** trimitic; generative hyphae bearing clamp connections, occasionally with simple septa; all the hyphae IKI–, CB+; tissues darkening in KOH. **Pellis**: Pellis cells regularly arranged into a palisade; terminal cells clavate, luminous yellow to yellowish-brown, thick-walled, occasionally expanded at the apex, moderately amyloid at maturity, 40–70 × 5–13 μm. **Context** generative hyphae colourless, thin-walled, with clamp connections, occasionally branched, 2–4 µm in diam; skeletal hyphae dominant, pale yellowish-brown, thick-walled to subsolid, frequently branched, interwoven, 3–5.5 µm in diam; binding hyphae abundant, pale yellowish-brown, thick-walled with a narrow lumen to subsolid, frequently branched, tortuous, interwoven, 1–3 µm in diam. **Tubes** generative hyphae colourless, thin-walled, mostly bearing clamp connections, occasionally with simple septa, occasionally branched, slightly swollen at the distal end, 1.5–3 μm in diam; skeletal hyphae dominant, pale brown to distinctly brown, thick-walled with a medium or narrow lumen to subsolid, frequently branched, strongly interwoven, 2–4.5 μm in diam; binding hyphae brownish-yellow, thick-walled to almost solid, frequently branched, interwoven, 1.5–2.5 μm in diam. Basidia barrel-shaped, yellowish to pale brown, with a clamp connection and four sterigmata, 12–18 × 9.5–13 µm; basidioles pear-shaped, 9–16 × 8–12 µm. **Basidiospores** mostly ellipsoid at maturity, truncate, yellowish to pale brown, IKI–, CB+, double-walled, exospore smooth, endospore with coarse echinulate, (8–)8.5–9 (–10) × (4.2–)5.5–6.5(–7) µm, L = 8.82 μm, W = 5.65 μm, Q = 1.52–1.60 (n = 60/2, with the turgid vesicular appendix excluded); (8.3–)9–10.2(–11.5) × (4.5–)5–6(–7) µm, L = 9.85 μm, W = 5.77 μm, Q = 1.68–1.72 (n = 60/2, with the turgid vesicular appendix included).

##### Type of rot.

a white rot.

##### Additional specimens examined.

CHINA. Guangdong Prov., Zhanjiang, Dianbai, on living trees of *Casuarina equisetifolia*, 4 October 2015, Dai 16337, Dai 16338, Dai 16339, Dai 17892, Cui 16370, Cui 16376 and Cui 16377 (BJFC!).

## Discussion

The two new *Ganoderma* species were found on living trees of *Casuarina equisetifolia* from the southeast coast of China. However, they are quite different from each other in morphology. Their main morphological differences are presented in Table 2. Phylogenetically, the two new species grouped together with some other laccate *Ganoderma* species in a well-supported clade.

**Table 2.** Morphological differences between the two new *Ganoderma* species collected on *Casuarina* from China.

Species	Pileal surface	Context	Cuticle cells	Shape of basidiospores	Size of basidiospores
*G. angustisporum*	reddish brown to dark brown	homogeneous, black melanoid band present	thin-walled, septate	almond-shaped	(8–)9–10.5(–11) × (3.5–)4–5 µm (with the turgid vesicular appendix excluded) (8–)9–11.3(–12) × (3.8–)4–5.2 µm (with the turgid vesicular appendix included)
*G. casuarinicola*	reddish brown	not fully homogeneous, black melanoid band absent	thick-walled to subsolid, non-septate	ellipsoid	(8–)8.5–9 (–10) × (4.2–)5.5–6.5(–7) µm (with the turgid vesicular appendix excluded)(8.3–)9–10.2(–11.5) × (4.5–)5–6(–7) µm (with the turgid vesicular appendix included)

In the phylogenetic tree inferred from ITS, EF1-α and RPB2 sequences, *G. angustisporum* clustered together with *G. boninense*, *G. ryvardenii* Tonjock & Mih and *G. zonatum* Murrill, these four species forming a strong support (BS = 100%, BPP =1.00; Fig. 1) lineage and could be distinctly separated from each other in the tree. Morphologically, all these four species produce laccate and sessile basidiomata, but *G. angustisporum* has the narrowest basidiospores amongst so far accepted *Ganoderma* species. *Ganoderma boninense* is another species producing relatively narrow basidiospores, but its basidiospores (8.7–12.8 × 4.7–6 μm, Zhou et al. 2014) are slightly wider than *G. angustisporum*. Moreover, its pileal surface ranges from orange to reddish-brown, even to fuscous or almost black and it lacks concentric growth zones in the context. *Ganoderma ryvardenii* differs from *G. angustisporum* by its larger pores (2–4 per mm), reddish basidiomata with waved margin and wider basidiospores (10–13 × 6–8 μm; Kinge and Mih 2011). *Ganoderma zonatum* mainly differs by larger basidiospores (10–12 × 5.3–6.3 μm, Zhou et al. 2014) and the absence of black melanoid band in the context.

In the phylogenetic tree, we obtained *G. casuarinicola* as sister to *G. enigmaticum* M.P.A. Coetzee, Marinc. & M.J. Wingf., a species described from South Africa, but morphologically, *G. enigmaticum* can be easily distinguished from *G. casuarinicola* by its golden yellow pileal surface without furrows and narrower basidiospores (8–11 × 3.5–6 μm, Coetzee et al. 2015). These two species gathered together with another South Africa species *G. aridicola* J.H. Xing & B.K. Cui, but *G. aridicola* is a sessile species with dark brown to black pileal surface, while *G. casuarinicola* has a reddish-brown pileal surface. Besides, *G. casuarinicola* has smaller basidia than *G. aridicola* (15–25 × 8–11 μm, Xing et al. 2016). Morphologically, *G. casuarinicola* resembles *G. tsugae* Murrill in having a reddish-brown pileal surface, white pore surface, similar wide ellipsoid basidiospores and lacking the black melanoid band in the context, but *G. tsugae* mainly differs by the absence of a light yellow layer under the laccate crust and concentric growth zones in the context (Zhou et al. 2014) and they are distinct from each other in the phylogenetic tree (Fig. 1). Besides, *G. tsugae* grows exclusively on conifers, especially on *Tsuga*, *Abies* and *Larix*, while *G. casuarinicola* occurs on hardwoods.

In conclusion, both morphology and phylogeny inferred from the combined ITS, EF1-α and RPB2 sequences support that the specimens, collected on living trees of *Casuarina equisetifolia* from the southeast coast of China, are two new species within the *Ganoderma* genus.

## Supplementary Material

XML Treatment for Ganoderma angustisporum

XML Treatment for Ganoderma casuarinicola
